# 

**Published:** 1891-01-24

**Authors:** 


### NOTICE TO CORRESPONDENTS.

All MS., Letters, Books for Review, and other matters intended for the Editor should be addressed The Editor, The Lodge, Porchester Square, London, W.

The Editor cannot undertake to return rejected M.S., even when accompanied by stamped directed envelope.

**Notice to Advertisers and Subscribers.**

All Advertisements, Orders for Copies of The Hospital, and Business Communications should be addressed to The Publisher, *not to the Editor*, The Hospital, 140, Strand, W.C.

The Cost of Subscription, post free, is as follows:—Per week, 1½d.; per quarter, 1s. 8d.; per half-year, 3s. 3d.; per annum, 6s. 6d. Foreign:—Per annum, 8s. 8d.; payable, in all cases, in advance.

**Bound Volumes of "The Hospital."**

Vol. VI., containing full page portraits of T.R.H. the Prince and Princess of Wales, and Miss Florence Nightingale, is still obtainable.

Vol. VII. and all the earlier volumes are also for sale.

Orders will be received for Vol. VIII., 4s. post free. Cases for binding may be had of the Publisher, at 1s. 6d. each.

**To Residents, Secretaries, and Matrons.**

The Editor will be glad to have the Regulations and each Report as issued by Asylums, Hospitals, Convalescent and Nursing Institutions, as well as those of other Charities, and an early copy in duplicate of all Appeals and Festival Notices, etc., addressed to *The Editor, The Lodge, Porchester Square, London, W.* Any superintendent, secretary, matron, or other chief official who regularly sends such information may be registered, on application to the Publisher, to receive free of cost a cover for binding The Hospital in half-yearly volumes.

### BURDETT'S HOSPITAL ANNUAL

May be obtained of the Publisher, The Hospital Offices, 140, Strand, W.C., price 2s. 6d. limp boards, or 3s. 6d. cloth. All orders must be accompanied by a remittance. (Note: The edition for 1891 will not be published until April, the current number containing latest information procurable to that date).

**Resident Medical Officers.**

### General Charities.

[This Column is devoted to an account of work of charitable institutions other than Medical Charities. The Editor will be glad to receive reports or news of work at 140, Strand. *Philanthropy* should be plainly written on the envelope.]

The committee of **THE EAST-END MOTHERS' HOME** appeal earnestly for additional funds to enable them to carry on their work, a description of which we hope to give in a future issue. Donations and furniture and clothing will be thankfully received by the Lady Superintendent, Mrs. Ashton Warner, at the Home, 393, Commercial Road, E.

The Committee of the **BRITISH ASYLUM FOR DEAF AND DUMB FEMALES,** Clapton, appeal for donations and subscriptions in order to meet increasing demands on them. The objects of the institution are to educate, give technical training to, and provide a home for deaf mutes over ten years of age. Donations will be gratefully acknowledged by the treasurer, Mr. Pascoe C. Glyn, or by the secretary, Mr. W. T. Hillyer, 27, Red Lion Square, W.C.

The Secretary to the **NATIONAL REFUGES FOR HOMELESS AND DESTITUTE CHILDREN** appeals for funds on behalf of 1,000 destitute boys and girls, who are maintained in their homes and ships, and who come from all parts of London and the provinces. Fifteen pounds will keep a child in food and clothing for one year; £1 5s. for a month; and 6s. 3d. for a week. For the first ten of the fifty, or nearly fifty, years of the operations of the Society, the labours of the Committee were confined to the notorious district then known as the Rookery of St. Giles. It was then found impossible to elevate permanently the lives of the little waifs and strays of this or any other district without removing them entirely from the surrounding of a neighbourhood where they were associated with nothing but vice and crime. By means of the various homes and of the training-ships which the Society has from time to time been enabled to establish this desirable object has been accomplished, and out of the 12,000 boys and girls received, more than 11,000 have been placed out in life to earn an honest livelihood in England or in the Colonies, and the Mercantile Marine, the Royal Navy, and the Army. In other words, this means the rescue of useful lives from degradation, vice, misery, pauperism, and the ranks of the criminal classes. There can be no more practical way of lessening the poverty and misery that is so rife in our midst than by ministering to the wants of the young. This society has proved by its work how much can be done to reduce the numbers of wasted, and worse than useless, lives that must ever be found in our large cities if we can only get at these lives before they have become hopelessly wedded to vice and crime. The resources of civilisation may prove inadequate to cope with confirmed vice, though that, we are glad to say has yet to be proved, but that they are able to rescue the young has been most clearly demonstrated by the acknowledged seccess of this institution. Donations to help them in their good work will be acknowledged with gratitude on behalf of the committee by Mr. William Williams, 164, Shaftesbury Avenue, W.C.

We are sorry to learn from Mrs. Kitto that she is under the necessity of appealing for aid to carry on the work which she started some eighteen months ago in the neighbourhood of the Haymarket for the rescue of such unfortunate women as are willing to abandon their wretched life. Without more funds it will be necessary to close the temporary resting place in Norris Street, where there are seven beds, and from which during the past year and a-half forty-six women have been sent to respectable homes or been placed in service. The house is neither a refuge nor a penitentiary, but merely a temporary shelter where a woman can remain till the committee or matron can discover the best way to help her. Some have been placed successfully in a dress-making home, others in "long homes," and the matron keeps touch with them after they have left Norris Street, so that the committee can gauge how far their efforts have been successful. Occasionally the police bring girls to Norris Street, sometimes an agent of the Church Mission, who has been working for Mrs. Kitto's committee, and many come from the Lock Hospital. One of the most satisfactory of the features of the work which is done by the committee in an unostentatious manner is the absence of restraint and of any attempt to force religion down the throats of people least in a condition to submit to it, which only encourage a desire to escape into freedom, even though it be a freedom of the least desirable nature. Mrs. Kitto's experience is that those girls who have been kept most strictly under religious and other forms of restraint are only too ready to avail themselves of the first opportunity which presents itself for breaking forth into their old life. The aim is to make their lives while in their temporary resting place as homely and simple as possible by means of persuasion and moral influence. The committee are anxious to find a lady who will work as a paid agent and so supplement the efforts of the matron and are equally anxious for donations in order that the work may be carried on. One of the great pleas for somebody to come forward and lend a helping hand to this undertaking seems to us to lie in the fact that the door is open on the very spot of a neighbourhood where these poor women congregate. Any sums will be gratefully acknowledged by Mrs. Kitto, St. Martin's Vicarage, Charing Cross, W.C.

### Scraps and Gleanings.

Mr. John Pitts has bequeathed £500 to Worcester Infirmary.

An anonymous donation of £500 has been paid to the Hospital Sunday Fund.

The Company of Carpenters have voted a sum of £70 to the London Hospital.

Lincoln County Hospital reports an income sufficient to meet the expenditure.

Mr. Martyn has been appointed house surgeon to the Devon and Exeter Hospital.

The sale of Dr. Koch's lymph will shortly be entrusted to the various chemists in Berlin.

Donations to the amount of £600 have been made to the Sanitary Institute during the past year.

Miss Emma Abbott, of New York, has left between £200,000 and £400,000 to various charities.

The Earl of Pembroke has sent £100 to the Royal Hospital for Diseases of the Chest, City Road.

The Lord Mayor on Tuesday held a reception at the Mansion House in aid of the Royal Free Hospital.

The death is announced of Mr. George Gulliver, M.B., senior assistant physician at St. Thomas's Hospital.

The London Hospital has received from the Queen a gift of twenty pheasants for the use of the patients.

The Queen has sent twenty pheasants for the use of the in-patients of the Cancer Hospital (Free), Brompton.

The 128th election to the British Orphan Asylum, Slough, was held on the 13th inst.; 10 boys and 8 girls were elected.

Owing to the prevalence of measles, all the elementary schools in Worthing will be closed during the next fortnight.

The customary gifts in money and kind to the poor of Richmond, Yorks, have been distributed on behalf of Lord Zetland.

The Duke of Portland will preside at the Twelfth Festival Dinner of the East London Hospital for Children, Shadwell, on March 2nd.

The London County Council will shortly consider the question as to the advisability of erecting and maintaining a central morgue for London.

During the month of December, 28,205 tons of meat were delivered at the Central Smithfield Market. Of this quantity 11 tons were found to be unfit for food.

An examination for commissions in the Army Medical Staff will be held in London on February 9th and following days. There are 20 commissions to be filled.

On Tuesday, 20th Dec., Professor Victor Horsley, Fullerian Professor of Physiology, R.I., began a course of nine lectures on "The Structure and Functions of the Nervous System."

The Lord Mayor has been appointed chairman of the Council of Almoners of Christ's Hospital under the new scheme. Mr. Walter Vaughan Morgan has been chosen as treasurer.

Lord Reay has consented to preside at the next festival dinner in aid of the funds of University College Hospital, to be held at the Whitehall Rooms, Hotel Métropole, on Tuesday, April 28th.

Owing to the accidents and failures that have happened in the treatment of consumptive patients by Dr. Koch's system, the patients in the Vienna hospitals have begun to refuse to submit themselves to inoculation.

The Queen has caused ten brace of pheasants to be sent for the patients of the Consumptive Hospital, Brompton, and the same number for those of King's College Hospital, and of the Seamen's Hospital Society (Dreadnought).

A verdict of accidental death, supplemented by a rider censuring the promoters of the entertainment, was returned by a coroner's jury at Leeds in the inquest upon the nine children who were burned to death during a New Year's fête.

Dr. Wendt, of New York, who was the first medical man to inoculate a black man with Dr. Koch's lymph, has found that in the case of coloured persons the effect of the remedy is much more violent, even if applied in very small doses.

The Committee of Management of the Hospital for Epilepsy and Paralysis, issue a Christmas appeal for funds to carry on their work. In 1889 155 in-patients were under treatment, and the "attendances" of out-patients showed an increase of 757 over the average of the past six years.

In the Hospital Sunday collections, St. Jude's Church, Kensington, keeps its place at the head of the list with a collection of £1,258 3s. 3d., this being the highest on record. St Michael's, Chester Square, follows with £1,106 3s. 6d., and then comes Christ Church, Lancaster-gate, with £1,016 17s. 6d.

A physician, named Bors, of Kaschau, in Hungary, claims to have discovered a remedy for diphtheria. Only two and a-half per cent. of the cases treated with Dr. Bors' remedy were fatal during a recent epidemic, while of the cases treated by other means the mortality was seventy-five per cent.

The annual general meeting of the British Medical Benevolent Fund was held on the 13th Dec., Sir James Paget, the president, in the chair. The report of the committee, which was adopted, showed that legacies amounting to £9,490 had been received and invested, and £2,020 was distributed in annuities. In subscriptions and donations £1,785 has been received, and £1,768 bestowed in grants to meet urgent cases of distress.

The annual full dress ball in aid of the funds of the Sunderland Infirmary took place in the Town Hall on January 6th. On several previous occasions the ball has been for the benefit of the Children's Hospital. That institution, however, has now amalgamated with the Infirmary, the usefulness of which has thereby largely increased. The ball last year realised over £180. This year the function was an even greater social success, and the financial result, it is hoped, will be correspondingly increased.

### THE STUDENT OF MEDICINE.

[All communications for this Supplement should be addressed to THE EDITOR of THE HOSPITAL, at 140, Strand, with the words, "Students' Column," written in the left hand top corner of the envelope.]

### APPOINTMENTS.

At St. Bartholomew's Hospital, Holbert Jacob Waring, M.B., L.R.C.P.Lond., M.R.C.S.Eng., has been appointed assistant house surgeon. At University College Hospital, G. B. M. White, M.B., B.Sc., F.R.C.S., has been appointed surgical registrar. At Westminster Hospital, A. M. Cato, L.R.C.P., M.R.C.S., has been appointed senior house physician, and F. B. Betts, L.R.C.P., M.R.C.S., late senior house surgeon, has been appointed junior house physician. At the Smithdown Road Workhouse, Toxteth, J. R. A. Bennett, M.R.C.S., has been appointed junior assistant medical officer, and J. W. Draper, L.R.C.P.Lond., M.R.C.S., senior assistant medical officer. At the Borough of Portsmouth Lunatic Asylum, Walter Reyner Brunton, L.R.C.P., M.R.C.S.Eng., has been appointed assistant medical officer. At the General Infirmary, Birmingham, Henry William Jacob, M.A., M.D., has been appointed assistant house surgeon. At the East Riding Lunatic Asylum, Beverley, Archibald Robertson Douglas, L.R.C.P., L.R.C.S.Edin., has been appointed assistant medical officer. At the Eveline Hospital for Sick Children, Southwark Bridge Road, Arthur H. Cheatle, M.R.C.S., L.R.C.P., has been appointed junior resident medical officer. At the Royal Infirmary, Newcastle-on-Tyne, W. Selby Plummer, M.B., B.S.Dunelm, has been appointed house surgeon. At the Seamen's Hospital, Greenwich, H. Mathew Spencer, M.A., M.B., C.M.Camb., has been appointed resident house physician.

### NOTES FROM THE SCHOOLS.

### Cambridge University.

The following degrees have been conferred: Bachelors of Medicine and Bachelors of Surgery—Walter Winslow, Gonville and Caius; Robert Stanley Thomas, Jesus; Jermyn Francis Gillett, Sidney Sussex; William Hubert Sale Fosberry, non-collegiate.

A course of instruction in practical hygiene is announced to be given by Mr. H. Robinson, assistant to the Professor of Chemistry, in the Chemical Laboratory. The course is to begin on April 17th, and is adapted to meet the requirements of the University and those of the General Medical Council for candidates seeking the diploma in public health.

### THE STUDENTS' BOOKSHELF.

"A Bench Book for Test Tube Work in Chemistry." By H. T. Lilley, M.A., London (Simpkin, Marshall, Hamilton, Kent, and Co., Paternoster Row). Perhaps of all the subjects that a medical student has to work at during his curriculum, none is a greater task for his memory than chemical analysis. A subject so full of detailed and complicated minutiæ is exceedingly perplexing to the mind, and it is one that cannot be mastered without tabulating the reaction of the various chemical bodies in a regular form. It is therefore with pleasure that we take up these dozen or so pages of the more common chemical reactions arranged all, more or less, in a tabular form, and in this way rendering a subject at first so complicated, at once tolerably clear. In this, the second edition of the work before us, a new table of solubility has been added, which is more graphic than those in general use, and may be used to illustrate the principles of some of the tests, especially the group tests for metallic radicles. The tables are devoted to qualitative analysis, and they commence with the solubility of the various bodies, thus at once dividing all substances into definite groups. Some remarks on the usual course of an analysis then follow, such as: Note the general appearance of the substance, whether coloured, metallic, amorphous, or crystalline, together with the size and shape of its crystals. Having dissolved the body, test the solution systematically for the basic radicle, confirming by the supplementary wet tests, and by the blowpipe tests, and so on. A table of the coloured salts is then given, and after this some general remarks with regard to the main principles to be applied in testing in general. The colour of the flame produced by burning some salts follow, and then the reactions from heating the body in a small dry tube. The next tables are those of the basic radicles in simple salts. They are divided into the following groups; Silver group, copper arsenic group, iron-zinc group, barium group. A table of supplementary wet tests is next given, and then the blow-pipe tests. We then come to the preliminary tests for acid radicles, and after this some further reactions for the bodies individually. The next table is devoted to the tests for the insoluble substances, after which there are some notes on basic radicles in mixtures, on the iron group, and on the separation of acid radicles. The final table deals with metals in mixtures, and this one is especially well arranged, giving each group clearly and distinctly. As stated in the preface, the object of the book is to provide a handy guide to practical work without diverting the attention of the student by undue reference to details of manipulation, which must, after all, be explained by the demonstrator, or learnt by experience. The tables appear to be very well suited for those engaged in practical chemistry, and would be a valuable addition to all students' chemical laboratories.

### PASS LISTS.

### Royal College of Physicians and Surgeons.

The following gentlemen passed the second examination of the board in anatomy and physiology at a meeting of the examiners on the 12th and 13th inst., viz.: Messrs. J. Prior, F. Horseman, R. Coates, and C. Oldfield, Yorkshire College, Leeds; J. W. Nayler, E. Bower. and D. William S. Muir. Queen's College, Birmingham; J. M. Hall and J. M'Connell, Queen's College, Belfast; E. A. Fraser, St. Mary's Hospital; F. J. Sadler, the Medical School, Oxford University; W. Barwise, Owens College, Manchester; J. F. Rudall, St. Thomas's Hospital; P. A. Dykes and W. P. Gwyn, the Medical School, Bristol; R. C. Badham, Sydney University and London Hospital; J. H. Saunders, Melbourne University and Mr. Cooke's School of Anatomy and Physiology; F. H. Langlands, Melbourne University and Middlesex Hospital; W. Anderson, the University of California; J. B. Winter, Guy's Hospital; J. Le Mare Bunch, University College; J. Evans, J. Sterry, and J. K. K. Benjamin, St. Bartholomew's Hospital; P. W. Paull and H. Huskinson, St. Thomas's Hospital; and C. M. Mathew, St. George's Hospital.

The following gentlemen passed in anatomy only, viz.: Messrs. A. H. Hardcastle, Yorkshire College, Leeds; J. Moses and W. S. Newton, London Hospital; C. C. Fenwick, St. Mary's Hospital and Mr. Cooke's School of Anatomy and Physiology; H. B. Emerson, Yorkskire College, Leeds, and Mr. Cooke's School of Anatomy and Physiology; J. W. Taylor, the Medical School, Bristol; G. H. Tomlinson, Queen's College, Birmingham; R. Alcock and H. C. Renshaw, Owens College, Manchester; F. R. E. Milman and A. E. Scott, the Middlesex Hospital; W. Allingham, St. George's Hospital and Mr. Cooke's School of Anatomy and Physiology; J. A. Crump, D. D. Brown, and A. G. Ewbank, St. Bartholomew's Hospital; F. A. Stephens, King's College Hospital; A. E. Couzens and A. Hewetson, St. Mary's Hospital; H. Shepherd and G. Davidson, St. Thomas's Hospital; E. J. Eedle, Charing Cross Hospital; and T. W. Goldney, Charing Cross Hospital and Mr. Cooke's School of Anatomy and Physiology.

The following gentlemen passed in physiology only, viz.: Messrs. T. B. Abbott, Yorkshire College, Leeds; F. D'Arcy M. Williams, the Medical School, Bristol; B. Watts, the Medical School, Sheffield; W. T. Talbot, the Boston University and London Hospital; P. R. Ash, A. Knowles, and W. Parkinson, Yorkshire College, Leeds; A. L. Knapman, J. Brown, and T. Clegg, Owens College, Manchester; T. P. Stokes, Sheffield and Mr. Cooke's School of Anatomy and Physiology; F. B. Cooper, the Medical School, Sheffield; E. G. Walls, Queen's College, Birmingham; A. G. Cooley, Durham University; V. J. Robin, Adelaide University and Mr. Cooke's School of Anatomy and Physiology; J. S. Williams, London Hospital; C. M. Spain, H. S. Taylor, and W. E. Kirby, University College; C. W. Grant-Wilson and J. S. Hudson, St. Thomas's Hospital; H. W. Joyce, King's College and Mr. Cooke's School of Anatomy and Physiology; E. C. Drake, Westminster Hospital and Mr. Cooke's School of Anatomy and Physiology; J. Thomas, St. Bartholomew's Hospital; F. H. Dayns, St. Mary's Hospital; F. H. U. Walker, St. George's Hospital and Mr. Cooke's School of Anatomy and Physiology; and J. W. Culmer, Guy's Hospital.

On the 14th and 15th inst.:—Messrs. C. E. Carpmael, A. H. Trevor, and C. H. Harding, Guy's Hospital; H. J. Finch, Westminster Hospital; E. S. Winter, P. L. G. Skipworth, and F. E. A. Webb St. Bartholomew's Hospital; T. G. Anderson, University of Adelaide and Mr. Cooke's School of Anatomy and Physiology; F. S. Beachcroft, Middlesex Hospital; C. H. Stewart, St. Thomas's Hospital; J. R. P. Phillips, St. Thomas's Hospital and Mr. Cooke's School of Anatomy and Physiology; F. R. S. Gaman, University College; D. S. Owen, A. J. Russell, A. E. Kennedy, and H. P. V. Wiggins, London Hospital; E. Reynolds and J. E. H. Davies, London Hospital and Mr. Cooke's School of Anatomy and Physiology; A. W. Quait, St. Thomas's Hospital; A. Murdock, St. Bartholomew's Hospital and Mr. Cooke's School of Anatomy and Physiology; J. A. Procter and J. P. Atkinson, King's College; A. W. Denny, Charing Cross Hospital; A. H. M. Hood and H. Witham, Westminster Hospital; and J. D. Seller, St. Mary's Hospital.

The following gentleman passed in anatomy only:—L. M. Breton and H. Knight, St. Thomas's Hospital; H. T. Jenkins, University College; W. J. Burroughs and G. E. Lockyer, Guy's Hospital; T. Smith and R. Bebb, London Hospital; F. B. Webster, Owens College, Manchester; W. H. Reed, Grant Medical College, Bombay, and King's College; J. J. Culmer and A. Alexander, Guy's Hospital; E. C. Peru, St. Thomas's Hospital; W. E. E. Powles, London Hospital; and D. D. Brown and C. W. Williams, St. Bartholomew's Hospital.

The following gentlemen passed in physiology only:—Messrs. S. R. Wright, Charing Cross Hospital; E. S. Chilcott, St. Mary's Hospital; W. S. Mayne, University College; F. E. Fielden, St. Bartholomew's Hospital; R. W. Dillon, St. Thomas's Hospital; J. B. Bettington and G. H. Steele, Guy's Hospital; H. W. Clarke, St. Mary's Hospital; G. R. Hannon, King's College Hospital; F. W. J. Goodhue, St. Thomas's Hospital; C. Corben, St. Bartholomew's Hospital; and P. C. Spark, Charing Cross Hospital.

### NOTICE TO CORRESPONDENTS.

A READER is requested to send name and address.

